# A CASE ARGUMENT FOR AGE DIVERSITY ON CAMPUS

**DOI:** 10.1093/geroni/igz038.1251

**Published:** 2019-11-08

**Authors:** Nancy Morrow-Howell, Edward Lawlor, Ed Macias, Emma Swinford, Jeffrey Brandt

**Affiliations:** Washington University, st. Louis, Missouri, United States

## Abstract

Washington University for Life is an initiative to increase age-diversity on campus, especially in degree and certificate programs. We have developed a case argument for educating people across the longer life course; and we identify positive outcomes for society, younger students, the institution, and for older adults themselves. These arguments do not include the reality that the supply of young students will be constrained, as this demographic press is not viewed as threatening by many U.S. universities. This argument is being presented to administrators, admission and career service personnel, and classroom instructors, who do not universally accept the benefits, much less the necessity, of increased age-diversity; and they often express resistance to disrupting traditional practices with only the well-being of older adults in mind. A full discussion of reasons that Washington University should move purposively toward age inclusivity is important to build consensus and identify opportunities for new practices.

